# Juvenile eosinophilic fasciitis: report of three cases with a review of the literature

**DOI:** 10.1186/1546-0096-13-S1-P161

**Published:** 2015-09-28

**Authors:** R Papa, P Nozza, C Granata, R Caorsi, M Gattorno, A Martini, P Picco

**Affiliations:** 1 Istituto Giannina Gaslini, Rheumatology, Genoa, Italy; 2 Istituto Giannina Gaslini, Anatomic Pathology, Genoa, Italy; 3 Istituto Giannina Gaslini, Radiology, Genoa, Italy

## Introduction

Eosinophilic fasciitis (EF), also called Shulman's syndrome or fasciitis-panniculitis syndrome, is an uncommon scleroderma-like disorder of unknown etiology, characterized by induration and thickening of skin and soft tissue, usually associated with peripheral eosinophilia. In childhood, EF is poorly characterized.

## Methods

A case series of all patients diagnosed with EF at our Department between 2011-2014 is reported. All cases with onset of EF before 18 year-old in PubMed-MEDLINE were reviewed: complete data were available for 16 pediatric cases only. These cases are compared to the present series and the adult form.

## Results

We report three cases of females (age range: 3-4 years) who developed a rapidly progressive motility impairment started from fingers, without cutaneous signs. They presented joint contractures, hepatosplenomegaly and generalized lymphadenopathy. Two of them developed acute complications (pharyngo-laryngeal incoordination and pericarditis). Laboratory investigations showed eosinophilia (range 1850-11670/mmc), increased erythrocytes sedimentation rate and hypergammaglobulinemia. The whole-body magnetic resonance imaging revealed thickening and hyperintensity of muscular fascia. A full-thickness biopsy showed inflammatory infiltration of the muscular fascia by mononuclear cells, confirming the diagnosis of EF. All patients were treated with oral prednisolone, plus methotrexate (1 patient) and cyclosporine (two patients), with partial benefit. Two years later, two of them show disabling outcomes despite intensive physiotherapy. In contrast with EF commonly described in adult patients, jEF is characterized by a very early onset. An association with trauma is missing, while history of infection was reported. The clinical presentation is dominated by a severe articular involvement that is prevalent in respect to the typical skin changes observed in adults. A systemic involvement (hepatosplenomegaly, lymph nodes enlargement) is more frequently present. Moreover, the disease course in children appears to be more severe.

## Conclusions

Juvenile EF may have onset with progressive motility impairment at upper extremities, without skin abnormalities, and may show systemic inflammatory involvement. Juvenile EF requires early recognition in order to start appropiate treatments aimed to prevent acute complications and long-term disabling outcomes.

